# Welfare assessment of broiler chickens at live bird market of Chattogram in Bangladesh

**DOI:** 10.5455/javar.2024.k832

**Published:** 2024-09-30

**Authors:** Md Ridoan Pasha, Mohammad Belayet Hossain, Amir Hossan Shaikat, Minhazur Rahman, Mohammad Rashedul Alam

**Affiliations:** 1 Department of Physiology, Biochemistry and Pharmacology, Chattogram Veterinary and Animal Sciences University, Chattogram, Bangladesh; 2 Department of Environmental Science, University of Chittagong, Chittagong, Bangladesh

**Keywords:** Broiler chickens, catching, lairage, live bird markets, stocking density, welfare

## Abstract

**Objective:**

This study aimed to assess the welfare conditions of broiler chickens in the live bird markets (LBMs) in Bangladesh.

**Materials and Methods:**

A total of fifty broiler outlets were studied in 10 LBMs of Chattogram, Bangladesh. A total of 10 chickens were observed to check the welfare issues during slaughter from each outlet (*N* = 500). The data were collected using a structured questionnaire method through interviews of the vendors and observation of the lairage and slaughter practice.

**Results:**

The study revealed that the stocking density was significantly higher in cage-type lairage than in floor-type (*p* < 0.05). The feeding and drinking areas for the chickens were significantly but negatively correlated to the stocking density. The duration between unloading of broiler chickens at LBMs and feeding or drinking could exceed 5 hours in 22% of outlets. The mortality was significantly higher in the bigger outlets than the smaller outlets (*p *< 0.05). During pre-slaughter handling, the one-wing grasping method was practiced more in the bigger outlets (*p *< 0.05) whereas the feet grasping method was used more in the smaller outlets (*p *< 0.05). Moreover, the knives used to slaughter the chickens were not sharpened daily in 76% of outlets.

**Conclusion:**

This study indicated that the broiler chickens in the LBMs of Chattogram had to face many stress episodes at different stages at their penultimate moments—from lairage to slaughter—which led to poor welfare conditions and exacerbated the suffering of chickens.

## Introduction

Bangladesh is one of the most densely populated countries in the world. The population is more than 164 million and it is increasing rapidly [1]. The poultry marketing and slaughter system of Bangladesh was not established to maintain the proper international standard [2]. The poultry markets are expanding rapidly in the city because of the rise in demand for chicken, particularly broiler chickens. In the traditional bazaar, there are live bird markets (LBMs) where chickens are often sold alive. Commercial farms have been developing a “broiler meat chain” over the past few years, where broiler chickens will be killed, processed, and sold in superstores with different cut varieties. However, the acceptance of this processed meat has divided perceptions among the general classes of people [3]. Moreover, as Bangladesh is a Muslim-majority country, the slaughter of animals in LBMs has its roots in religious practices to ensure halal food [4].

Chattogram is one of the largest cities in Bangladesh. Being an industrial and business hub, this city is the habitat of more than 5.3 million people and broiler chickens are one of the key meat suppliers for this population [5]. The broiler chickens are usually reared at the farms on the periphery of the city and then are harvested from the farms and transported to the LBMs by the middlemen. The chickens are then kept in cages or on floors in the shops until they are slaughtered after performing a religious call. No pre-slaughter protocols, including stunning, are followed. Generally, the chickens are left to bleed, and after that, the carcasses are processed according to the choice of the consumers.

In the well-established broiler chickens’ slaughterhouses, the chickens are stunned, shackled, and bled. Therefore, the welfare issues of broiler chickens in a modern or established slaughterhouse are associated with these factors and stages like the duration of transport, death on arrival, plumage conditions, injuries and bruises, and post-slaughter conditions of the carcass and these are thoroughly investigated in different studies to address various welfare issues [6–9]. However, as the traditional LBM system is different from the slaughterhouse practice, many of the stages in slaughterhouses are absent here. In addition, as all humans are responsible for establishing the five freedoms of animals, the welfare of broiler chickens at the LBMs needs to be established too [10]. However, the poor welfare issues and malpractice—that can hamper good welfare conditions for broiler chickens—at lairage and during slaughter in the LBMs of Bangladesh are not been properly addressed in previous studies.

Therefore, this work aims to address and assess the existing conditions at LBMs by evaluating various lairage practices—the rearing area, the stocking density, the feeding and drinking space per chicken, the feed and water source, and mortality—and the slaughter events—handling of the chickens before slaughter, and management of slaughtering utensils—to find out the probable breach in the welfare of broiler chickens at Chattogram metropolitan area.

## Materials and Methods

### Ethical approval

This study was undertaken at the Department of Physiology, Biochemistry and Pharmacology of Chattogram Veterinary and Animal Sciences University (CVASU). The conduct of the experiment was approved by the CVASU Ethics Committee on 21 July 2020 [CVASU/Dir(R&E)EC/2020/169/6].

### Market and shop selection

There are a good number of permanent and temporary LBMs in Chattogram metropolitan city (22°13’ and 22°27’ north latitudes and between 91°40’ and 91°53’ east longitudes). Among all the permanent markets, a total of 10 LBMs across this city were selected for the present study. From each market, five broiler shops were selected where broiler chickens were sold and slaughtered (*N* = 50). The study was conducted between March 2019 to May 2019.

### Data collection procedure

The data focusing on the welfare of the broiler chickens at these broiler shops were collected through interviews with the vendors and the observation of the other factors. The interview was performed based on a structured questionnaire. On the other hand, the observation was performed on the broiler shop condition, especially the lairage and lairage facilities, and the welfare issues during slaughter practice. The terminologies used in the study for the assessment of market, shop, slaughter facility and chickens are compiled in the Table 1. The LBM system differs from the slaughterhouse system; practiced in developed countries. Hence, the welfare assessment was performed based on some principles, criteria, and measures adapted to this live bird market system (Table 2).

**Table 1.** Definitions of terminologies used in this article.

Terminologies	Definitions
Live bird market	The open marketplace where meat-producing birds are sold
Vendor	The poultry shop owner who sells broiler chickens
Broiler shop	The place is in a market where broiler chickens are kept in lairage, sold, slaughtered, and processed.
Unloading	The process of transferring the chickens from the vehicle to the bird shops
Lairage or lairage system	The place—either floor or cage—where meat-producing birds are kept for showing to the consumers
Knife	The sharp tool used to slaughter the broiler chickens
Grasping	Catching and holding the broiler chickens to show the consumers before slaughter
Ventilator	A small window-like open space in the wall to help in aeration
Exhaust fan	A fan was kept in the ventilator to facilitate the ejection of bad gases and hot air from the broiler shop
Mortality rate	The number of deaths of broiler chickens in the lairage

### Interview of the vendors

A structured questionnaire was prepared after a pilot study. “Welfare Quality Assessment Protocol for Poultry (2009)” produced by Welfare Quality^®^ was used as a base to assess the housing conditions at the broiler shops [11]. For other factors, the shop owners or vendors were asked regarding general information like their business experience, total sales per day, death of chickens per day, unloading time of chickens, duration between feed supply and unloading (to assess the hunger period), duration between water supply and unloading (to assess the thirst period), feed source, water source. In these broiler shops, broiler chickens were also slaughtered within the shop premises. Therefore, the shop owners were also asked about the frequency of the sharpening of the knives.

### Shop observation

In the shops, the chickens were usually kept in cages or floors for lairage. The cage area and floor area were measured and were used to measure the stocking density. The numbers of feeders and drinkers were counted and feeding and drinking spaces were measured using measuring tape. The cages were observed to find out whether any sharp objects or ends were present which might cause injury to the birds. In the floor system, the litter condition was graded according to the assessment protocol (Table 3). The plumage cleanliness of the chickens was also observed and graded. The shops were observed whether there was the presence of ventilators and exhaust fans within the shops.

**Table 2.** List of the principles, criteria, and measures that were assessed for animal welfare evaluation in the live bird market (Adapted from “welfare quality assessment protocol for poultry (2009)”) [11].

Principles	Criteria	Measures	Measurement method
Good feeding	Prolonged thirst	The duration between unloading and water supply	Shop based measurement
		Drinker space (Total drinking area)	Lairage based measurement
	Prolonged hunger	The duration between unloading and feed supply	Shop based measurement
		Feeding space (Total feeding area)	Lairage based measurement
Good housing	Stocking density	Total number of chickens, multiplied by an average weight and divided by the area measurement	Lairage based measurement
	Aeration	Presence or absence of the exhaust fan in a shop	Shop based measurement
		Presence or absence of ventilators in a shop	Shop based measurement
	Litter condition	Graded according to “Welfare Quality Protocol”	Lairage-based measurement (only floor)
Good health	Mortality	Counting the number of dead chickens in a shop	Shop based measurement
Appropriate behavior	Good human-animal relationship	Observing the grasping method of chickens going to be slaughtered.	Ten chickens are observed in each shop.

**Table 3.** Litter quality (Adapted from “Welfare Quality Assessment Protocol for Poultry, 2009”) [11].

Classification	Description
0	Completely dry and flaky, i.e. moves easily with the foot
1	Dry but not easy to move with the foot
2	Leaves imprint of foot and will form a ball if compacted, but the ball does not stay together well
3	Sticks to boots and sticks readily in a ball if compacted
4	Sticks to boots once the cap or compacted crust is broken

### Observation of birds before and after slaughter

The handling of the chickens by the shop owners during the sales event was observed. The chicken-holding technique of the shop owners was also noted. Moreover, the average weight of the chickens in a certain setting—either floor or cage or both—was collected during this time. A total of 10 chickens were observed in each shop to assess this parameter (*N* = 500). The weight of 10 chickens was noted to calculate the average weight which was used to measure the stocking density.

### Statistical analysis

The questionnaire and observation data were input in Microsoft Excel 2013. After that, the data were analyzed using the statistical software STATA-14 (StataCorp, Texas, USA). The descriptive study was performed for the lairage and slaughter information related to the chickens’ welfare. The relationships between the total sale of chickens per day in a shop with daily mortality rate, and the grasping method during catching of the chickens before slaughter were assessed by *t*-test. The difference in the stocking density between two lairage systems—floor and cage—was revealed by the chi-square test. Pearson correlation test was performed to find out the relationship between the stocking density, feeding spaces, and drinking spaces in the lairage systems. The relationship having *p* < 0.05 was taken as significant for all the analyses.

## Results

### General management in lairage and slaughter practice

Among the fifty broiler chickens’ shops, 26 had cages, 14 had floors whereas 10 shops had both types of lairage. In the lairage, around 50% of shops provided less nutritious sale center feed or broiler starter feed. The chickens are generally unloaded from the transport vehicle into the shops. The vendors supply the feed and water to those chickens. In 54% of cases, the provision of feeds and water is not immediate and in 22% of cases, the vendors take 5 h or more to provide these necessities. The knives used for sharpening were sharpened daily only by 24% of vendors (Table 4).

**Table 4.** Descriptive data regarding management in live bird shops at LBMs at Chattogram (*N* = 50).

Variables	Category	Frequency (%)	95% CI
Lairage	Cage	26 (52)	37.4–66.3
	Floor	14 (28)	16.2–42.5
	Both	10 (20)	10–33.7
Litter condition (*N* = 24)	0	2 (8.3)	1.02–27
	1	5 (20.8)	7.1–42.1
	2	8 (33.3)	15.6–55.3
	3	6 (25)	9.8–46.7
	4	3 (12.5)	2.6–32.4
Types of feed in lairage	Sale center feed*	23 (46)	31.8–60.7
	Broiler starter	2 (4)	0.5–13.7
	Broiler grower	25 (50)	35.5–64.5
Source of water	WASA	21 (42)	28.2–56.8
	Tube Well	26 (52)	37.4–66.3
	Pond	3 (6)	1.3–10.6
Supplements in water (After unloading to relieve the stress of chickens)	Not added	30 (60)	45.2–73.6
	Vitamin C and Lemon juice	19 (38)	24.6–52.8
	Tamarind water	1 (2)	0.05–10.6
Immediate task after unloading	Water supply	12 (24)	13–38.2
	Feed supply	2 (4)	0.5–13.7
	Both	36 (72)	57.5–84.8
Duration between unloading and feeding/water supply	Immediate	23 (46)	31.8–60.7
	≤ 1 h	16 (32)	19.5–46.7
	≥ 5 h	11 (22)	11.5–36
Ventilators	Absent	22 (44)	30–58.7
	Present	28 (56)	41.3–70
Exhaust fan	Present	2 (4)	0.5–13.7
	Absent	48 (96)	86.3–99.5
Sharp objects in cage (*N* = 36)	Absent	32 (88.9)	73.9–97
	Present	4 (11.1)	3.1–2.6
Industrial sharpening of the knives	Everyday	12 (24)	13.6–38.2
	Not everyday	38 (76)	61.8–87

*It is a mixture of low-quality feed ingredients prepared in the feed shops at the market.

### Lairage management

In the lairage, the chickens are kept until slaughter which could be more than 1 day in some shops. Table 5 showed that a higher stocking density was maintained significantly higher in cages (in 72.2% of cages) than on the floors (in 12.5% of floors). On the other hand, the Pearson correlation coefficient analysis was computed to assess the linear relationship between stocking density, feeding area, and drinking area (Table 6). There was a significantly negative correlation between stocking density and total feeding area. The same phenomenon was found between stocking density and total drinking area. However, there was a positive and highly correlated relationship between the feeding area and the drinking area.

### Mortality

According to Table 7, in the lairage, the smaller shops (daily total sale is ≤100 chickens) encounter significantly higher mortality (≥1% in 65.2% shops) whereas, in bigger shops (daily total sale is >100 chickens), the mortality rate was lower (≥1% in 37% shops).

**Table 5.** The relationship between stocking density (kg/m^2^) and lairage type (*N* = 60).

Parameters		Stocking density		*p*-value
		≥30 kg/m^2^	<30 kg/m^2^	
Lairage type	Cage	26	10	0.00
	Floor	3	21	

**Table 6.** The relationship among stocking density (kg/m^2^), total feeding area, and the total drinking area in the lairage (*N* = 60) (Asterisk was put in the significant relationship at 5% level of significance).

Parameters	Stocking density	Feeding area	Drinking area
Stocking density	1.00		
Feeding area	−0.63^*^	1.00	
Drinking area	−0.61^*^	0.93^*^	1.00

**Table 7.** Relationship between daily mortality percentage with the daily sale in the broiler shops (*N* = 50).

Parameters		Mortality rate		*p*-value
		<1%/day	≥1%/day	
Total sale of broiler chickens	≤100/day	8	15	0.04
	>100/day	17	10	

**Table 8.** Relationship among sale, and chicken handling methods (*N* = 50).

Grasping method		Feet (Mean ± SE)	One wing (Mean ± SE)	Both wings (Mean ± SE)
Total sales/day	≤100/day	5.26 ± 0.9	3.22 ± 0.8	1.47 ± 0.5
	>100/day	1.96 ± 0.6	5.63 ± 0.7	2.22 ± 0.6
*p*-value		0.005	0.03	ns

### Pre-slaughter catching and handling

The broiler chickens are usually shown to the consumers before slaughter and weighed. During this period, the chickens are held either by feet, one wing, or both wings. It was observed that in smaller shops chickens were held mostly by feet whereas, in bigger shops, the vendors used to hold the chickens by one wing. In both types of shops, very few chickens were held using both wings (Table 8).

## Discussion

### Prolonged thirst and hunger

The broiler chickens are generally raised at the farm outside of the city and then brought to the LBMs. During this process, the chickens are generally left without water and feed. After reaching the LBMs, which can be any time between early morning to late night, the vehicle operators unload the chickens at the shops. Generally, the feeders and drinkers are cleaned during the closing hours of shops and markets. So, when the shop is closed at night there remains no water and feed at the troughs. In the morning, when the shop owner returns, he supplies the water and feeds the chickens. The duration between this unloading of chickens to the feed or water supply can be immediate (46%) if the vehicle can reach in the morning when the shops open (Table 4). The duration can exceed more than 5 h (22%); generally, when the vehicles carrying chickens reach late at night. This long duration of water and feed restriction along with the transport stress and environmental stress because of the hot and humid environment in Bangladesh can cause dehydration and fasting effects on chickens and cause other metabolic changes in the body which can ultimately affect the carcass or cause the death of chickens, which is reflected in the Table 7 [12–14].

### Feed type and supplementation in water

During the finishing period of broiler chickens reading, they are fed broiler grower feed. In the LBMs, all the chickens are not slaughtered at the same time. To keep the weight of the chickens, the vendors usually feed them “Sale Center Feed” which is a mix of low-quality feed ingredients prepared in the feed shops at the market. About 46% of vendors use this feed for the broiler chickens as it is cheaper than the broiler starter or grower feeds, which can be devoid of proper nutrition as these are not formulated properly (Table 4). To quench the demand, the body may break down the glycogen and protein pool from muscle and this factor is also a stressor for the chickens [15–16]. In addition, during summer, the temperature and humidity can go above the comfortable level for chickens. To alleviate thermal stress, supplementation of various nutritional products such as vitamin C, betaine, and selenium are found to be effective [17,18]. However, only 40% of vendors were supplementing their broiler chickens—especially vitamin-C powder—during the summer season (Table 4). This may cause stress on the broiler chickens and may lead to death in the lairage [8,9,19].

### Stocking density and its effects on feeding and drinking space

Among the 50 shops, 26 had cage type, 14 had floor type and 10 had both types of broilers chickens’ holding or lairage. Generally, the chickens are kept on the floor with deep litter—mostly sawdust—in the floor type whereas the cages are made of steel. It was found that the stocking density was significantly higher in the cages than in the floor (Table 5). Moreover, the drinking and feeding areas decreased significantly when the stocking density was high (Table 6). Generally, the vendors keep the feeder and drinker on a thumb-rule basis in the lairage. When they keep more chickens for sale, the average feeding space and drinking space gets reduced. Sometimes, they even reduce the number of feeders and drinkers to allow more chickens in the lairage. Generally, when the feeder number has increased, the visit to the feeders is also increased [18]. In a low stocking density, the chickens can consume more water [20]. In addition, higher stocking density especially in warmer conditions can induce multiple welfare issues like the fasting of chickens, injury, and fearfulness, and affect the physiology and behavior of chickens [21,22].

### Ventilation and litter condition

Broiler chickens require good aeration for their dissipation of heat. About 56% of broiler shops did not have any ventilators in the shops. About 96% of shops did not have any exhaust fan to relieve the hot air. Hence, the environment within the lairage and shop always remains hot. This hot temperature may cause thermal stress in broiler chickens, affect their physiology, and may also cause death [23,24]. In the floor-type lairages, the litter condition was also assessed. The litter condition was scored from 0 to 4 (Table 3). Conditions 2, 3, and 4 reflect poor litter quality and 70.9% of the floor had these types of lairage (Table 4). Poor litter may cause foot pad dermatitis, and hock burn and can increase the temperature in the shed [25]. In cage-type lairage, sharp objects were found in 11.1% of cages which may cause injury to the chickens (Table 4). The environment and lairage conditions of the broiler chickens in both settings—cages and floors—were not satisfactory.

### Mortality rate

In the LBMs, the death or mortality of chickens is one of the major welfare and economic concerns. It was found that the mortality rate was higher in the smaller shops (sales are less than or equal to 100 chickens/day) than in the bigger shops (sales are more than 100 chickens/day) (Table 7). Generally, the smaller shops have less space. The chickens are usually held in those shops for longer periods. During this time, the chickens face many stressors (handling stress, thermal stress, and so on) that may cause death [7,12].

### Pre-slaughter catching and grasping

A total of 500 chickens (10 chickens from each shop) were observed to assess their preslaughter handling. Chickens were held in three manners; holding by feet, holding by one wing only, holding by both wings. It was found that the shops with larger sale volume (>100/day) tend to use the one-wing grasping method significantly more than the shops with smaller sale volume (≤100/day) (5.63 ± 0.7 and 3.22 ± 0.8, respectively). On the other hand, the holding by feet was found significantly more in the smaller shops (5.26 ± 0.9) than in the bigger shops (1.96 ± 0.6) (Table 8). It can be caused because of the rush of sale as holding by one wing is quicker than the other methods and holding by feet is less quick. The wing is very sensitive to being injured. Rapid catching and rough handling can cause wing injury, fracture, bruises, and even death [8,9,26–28]. This is an important stressor to the chickens and a major welfare concern in the LBMs. In addition, the knives used for slaughter are needed to be sharpened every day. However, 76% of the vendors did not sharpen their knives—every day—using the industrial sharpener (Table 4). Though this study aimed to assess the welfare conditions of broiler chickens at LBMs in Bangladesh, an important limitation of this study is that it was conducted in only 1 metropolitan area—Chattogram—and assessed only 10 LBMs. Further research with a larger sample size incorporating more metropolitan and rural areas will help to better visualize the welfare conditions and investigate the welfare issues in the LBMs of Bangladesh.

## Conclusion

The goal of this research was to assess the welfare of broiler chickens at LBMs in Chattogram, Bangladesh. The LBMs have a lot of negative welfare concerns. The cage-based lairage system had a high stocking density, while lower stocking densities tended to have smaller feeding and drinking areas. In the shops with higher sales volumes, the catching was stressful and the one-wing technique was implemented more. This study has given a comprehensive understanding of the welfare conditions of broiler chickens in LBMs in Bangladesh. To identify all potential welfare concerns at LBMs and their effects, further study is necessary.

## List of abbreviations

CVASU, Chattogram Veterinary and Animal Sciences University; LBMs, Live Bird Markets.
